# Effect of standard light illumination on electrolyte’s stability of lithium-ion batteries based on ethylene and di-methyl carbonates

**DOI:** 10.1038/s41598-018-36836-9

**Published:** 2019-01-15

**Authors:** Gaspard Bouteau, Albert Nguyen Van-Nhien, Michel Sliwa, Nicolas Sergent, Jean-Claude Lepretre, Grégory Gachot, Iryna Sagaidak, Frédéric Sauvage

**Affiliations:** 1 Laboratoire de Réactivité et Chimie des Solides, Université de Picardie Jules Verne (UPJV), CNRS UMR 7314, 33 rue Saint Leu, 80039 Amiens, France; 2 Réseau sur le Stockage Electrochimique de l’Energie (RS2E), FR CNRS 3459, 33 Rue Saint Leu, 80039 Amiens, France; 3 Laboratoire des Glucides Université de Picardie Jules Verne (UPJV), CNRS UMR 7378, 33 rue Saint Leu, 80039 Amiens, France; 4 University Lille, CNRS, UMR 8516 - LASIR - Laboratoire de Spectrochimie Infrarouge et Raman, F-59000 Lille, France; 5 Laboratoire d’Electrochimie et Physicochimie des Matériaux et de Interfaces, Université de Grenoble Alpes, CNRS UMR 5279, 1130 rue de la piscine, 38402 Saint Martin d’Hères, France

## Abstract

Combining energy conversion and storage at a device and/or at a molecular level constitutes a new research field raising interest. This work aims at investigating how prolonged standard light exposure (A.M. 1.5G) interacts with conventional batteries electrolyte, commonly used in the photo-assisted or photo-rechargeable batteries, based on 1 mol.L^−1^ LiPF_6_ EC/DMC electrolyte. We demonstrate the intrinsic chemical robustness of this class of electrolyte in absence of any photo-electrodes. However, based on different steady-state and time-resolved spectroscopic techniques, it is for the first time highlighted that the solvation of lithium and hexafluorophosphate ions by the carbonates are modified by light exposure leading to absorbance and ionic conductivity modifications without detrimental effects onto the electrochemical properties.

## Introduction

Introduction of renewable energies amongst photovoltaics and wind prompts the hybridization with energy feedstock to alleviate intrinsic and unpredictable large-scale fluctuation in energy production. Electrochemical batteries and supercapacitors developments are strategic to stabilize the future electrical grid and to provide sufficient energy at demand for the end-users. One advantage to endorse their development is the large versatility in their design to accommodate high power -in and –out and/or promoting large energy storage. As positioned in longer-term perspectives, research to develop combined photovoltaic and electrochemical storage technologies is gaining interest either by introducing a photo-electrode inside the battery to reduce the power requirements for the battery recharge when placed under illumination^1–6^ or by introducing new concepts such as photosensitized battery electrode or semi-conducting electrode to combine solar energy conversion and chemical storage^7–13^. This latter approach was first observed on chalcogenide electrode in contact with sulfide electrolyte by Hodes in 1976 and pursued in the early 1980’s by Tributsch *et al*. highlighting a light-induced interfacial ion transfer in mixed electronic/ionic p- and n- type semi-conductors^14,15^. However, although this photo-reaction is made possible because of the longer carriers lifetime into the depletion layer, the very low value of the Debye length compared to the particle size strongly confines such an interfacial to the extreme surface of the particles, thus limiting its application to functional device^8^. We recently demonstrated using n-type anatase TiO_2_ particles in lithium technology that such asan impediment can be overcome by preferring a high dielectric constant semi-conductor to enhance the exciton dissociation, prolong the excited-state lifetimes and widen the Debye length, and secondly, by carefully synthetizing mesoscopic photo-electrodes made of nano-crystalline particles instead of micro-sized^13,16^.

So far, the different achievements reported in literature are mainly focused on the photoactive materials and the gain in performances into the electrochemical battery recharge as a result from the light absorption process. However, much less attention has been paid to the components stability upon cycling. In addition, although well-studied in darkness under electrochemical bias for different electrode materials^17–23^, the effect of prolonged light exposure on the stability of the most-common battery electrolytes has still not been investigated (ie. LiPF_6_ in a carbonates solvent mixture)^11,13^. Electrolyte stability in photoelectrochemical device is generally a key requirement to ensure long-term stability. For such a new concept of photo-rechargeable batteries, this rule is also proved as pointed out by Zaghib *et al*. and our group who recently demonstrated that electrolyte and semi-conductor/electrolyte interface is one important concern to address towards the development of more stable photo-batteries^11,13^. This work is discussing the intrinsic batteries electrolyte photo-stability under Air Mass 1.5 G conditions on the basis of NMR and mass spectrometry as well as on steady-state and time-resolved spectroscopies.

## Results and Discussions

**Figure 1:**
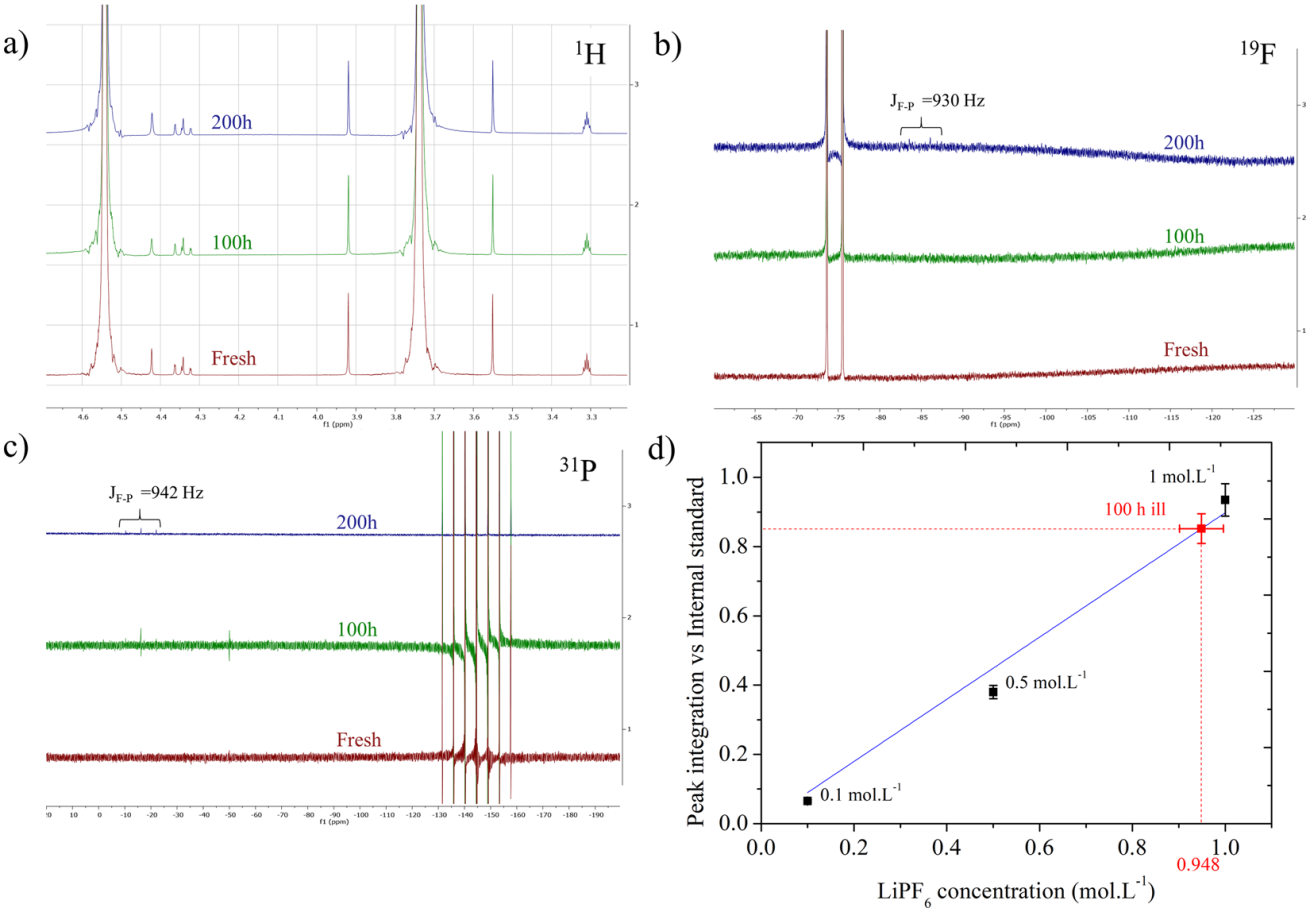
(**a**) ^1^H, (**b**) ^19^F and (**c**) ^31^P NMR spectra of 1 mol.L^−1^ LiPF_6_ EC/DMC 1/1 weight ratio before and after 100 and 200 hours illumination under A.M.1.5G conditions. (**d**) Quantification of LiPF_6_ concentration in the electrolyte after 100 hours of light soaking using ERETIC-NMR method (^19^F NMR). This figure reports the integration value of the fluor doublet compared to the internal standard (FEC) for different concentrations.

The chemical stability of the electrolyte before and after continuous illumination time, ie. 100 and 200 hours under A.M. 1.5 G conditions (100 mW/cm^2^) was first assessed by ^1^H, ^19^F and ^31^P NMR. As showed in Fig. 1a in the ^1^H spectra, prolonged light exposure does not contribute to any substantial degradation or modification of the EC/DMC ratio of the carbonates in the electrolyte based on the absence of any chemical shift or new peaks appearing. The chemical robustness was also confirmed by more sensitive techniques as gas chromatography/mass spectrometry (GC/MS) analysis of the different electrolytes showing the absence of any degradation byproducts (Fig. S1). The peak at t = 20.38 min is attributed to EC whereas DMC is hidden under the acetonitrile solvent front used for the analysis. This observation is in good agreement with the NMR analysis of electrolyte in contact with N719-sensitized LiFePO_4_ photoelectrode by Zaghib *et al*.^11^. For ^19^F and ^31^P analysis (Fig. 1b,c, respectively), which is related to the lithium hexafluorophosphate salt, no shift in the native doublet of the fluor or septet of the phosphorus is experienced after 100 and 200 hours of illumination. However, one can notice the onset of a small contribution as a doublet at 84.83 ppm (^19^F) and a triplet at −16.21 ppm (^31^P) with a coupling constant of 930 Hz and 942 Hz after 200 hours illumination, respectively. These new contributions are attributed to POF(OH)_2_ and POF_2_(OH) that have also been observed by Zaghib *et al*.^11^, which are stemming from LiPF_6_ hydrolysis in presence of water traces^19^. The amount of salt consumed by this reaction has been estimated using the ERETIC-NMR method (Electronic REference To access *In vivo* Concentrations). For this, fluoroethylene carbonate (FEC) was introduced as an internal standard for ^19^F NMR prior to the NMR measurements. The integration value of the FEC is compared to the one of the Fluor doublets for different concentrations to set the calibration curve. As a result, we quantified the LiPF_6_ concentration for the 100 hours illuminated sample to be ca. 0.948 ± 0.045 mol.L^−1^ (Fig. 1d), thus no or very little LiPF_6_ is consumed after 100 hours of illumination. For these experiments, one can conclude that neither the carbonate mixture nor the hexafluorophosphate lithium salt of the electrolyte are decomposed subsequently to light illumination, demonstrating its intrinsic photostability (i.e. in the absence of any electrode materials and electrical bias).

**Figure 2:**
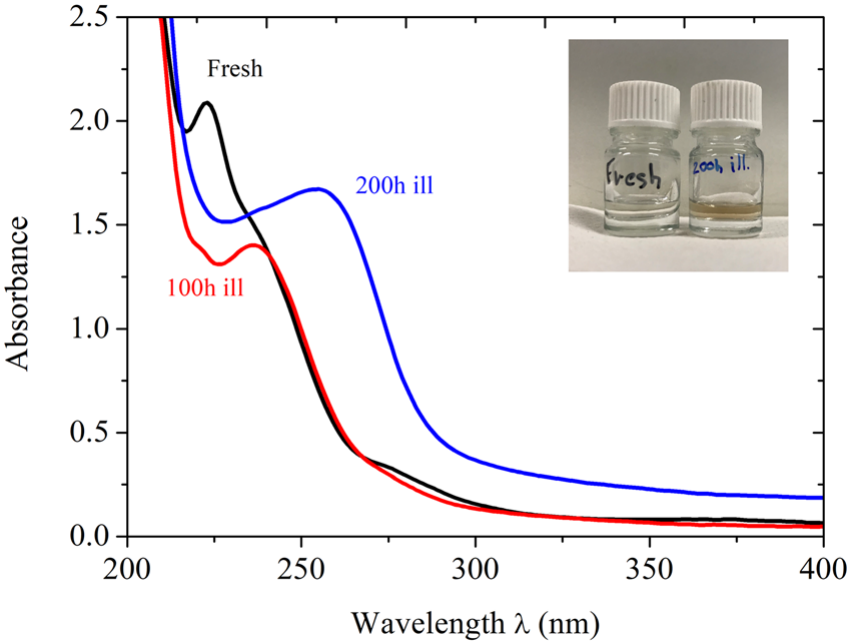
UV-Visible absorption spectra of fresh 1 mol.L^−1^ LiPF_6_ EC/DMC electrolyte (black curve), after 100 hours (red curve) and 200 hours (blue curve) of light illumination under A.M. 1.5G conditions. A picture of the electrolyte before and after 200 hours continuous light irradiation is presented in inset.

Interestingly, although any substantial electrolyte degradation is herein ruled out by both NMR and GC/MS analysis, the initially transparent colorless electrolyte tends to become yellowish upon increasing the illumination time (Inset Fig. 2). Figure 2 gathers the evolution of the UV-visible absorption spectra of the different electrolytes as a function of illumination time. Pristine electrolyte displays main optical absorption below 300 nm, E_(HOMO-LUMO)_ > 4.1 eV. Before any light irradiation (Fig. 2 black curve), the absorption spectrum shows a first band below 210 nm and four others between 220 and 400 nm which can be deconvoluted as follow: 223 nm, 237 nm, 275 nm and 370 nm. The deeper band in UV is the greater in absorption. After 100 hours of illumination, the absorption band at 237 nm remains comparable (Fig. 2, red curve). The main differences are the decrease in absorption of the band at 223 nm and the disappearing of the bands at 275 and 370 nm. By contrast, a significant modification in the whole acquired spectrum can be observed after 200 hours light soaking (Fig. 2, blue curve). Below 300 nm, the absorption spectrum shows only two main contributions, one broad at 255 nm and a second below 210 nm comparable with the two other samples. The yellowish color arises by the occurrence of a broad absorption tail above 300 nm. Unfortunately, more specific attribution of these different optical transitions requires molecular calculations such as by TD-DFT method which has never been led so far to the best of our knowledge. However, by comparing the different contributions of each electrolyte components, it is possible to attribute the four bands at <210 nm, 223 nm, 237 nm, and 275 nm to EC/DMC solvent mixture and the one at 375 nm to LiPF_6_/DMC (Fig. S2, Table 1). The addition of LiPF_6_ in EC/DMC solution induces a hypsochromic shift of the 230 nm and 246 nm bands on the one hand and an hyperchromic shift in the whole spectrum on the other hand (Fig. S2, Table 1). It also leads to the appearance of a small band at 375 nm and a tail until 400 nm which are also noticed for LiPF_6_ in DMC. We hypothesize the blue-shift and the increase of the absorbance to stem from intermolecular transitions between PF_6_^−^ and EC/DMC. Based on these results, the consequence of exposing the electrolyte to prolonged incident light is a noticeable molecular reorganization of the solvated LiPF_6_ species.

**Table 1:** Position and absorbance value of the different absorption bands for the different electrolytes reported in Fig. S2 and for the illuminated electrolytes reported in Fig. 2 (The contribution below 210 nm is not reported in the table).

	LiPF_6_ DMC		EC/DMC		LiPF_6_ EC/DMC		100 h illumination		200 h illumination	
	λ (nm)	Abs.	λ (nm)	Abs.	λ (nm)	Abs.	λ (nm)	Abs.	λ (nm)	Abs.
Band 1	/		230	0.41	223	2.03	223	1.36	/	
Band 2	/		246	0.21	237	1.49	237	1.39	255	1.67
Band 3	/		274	0.05	275	0.34	/		/	
Band 4	375	0.015	/		370	0.012	/		/	

**Figure 3:**
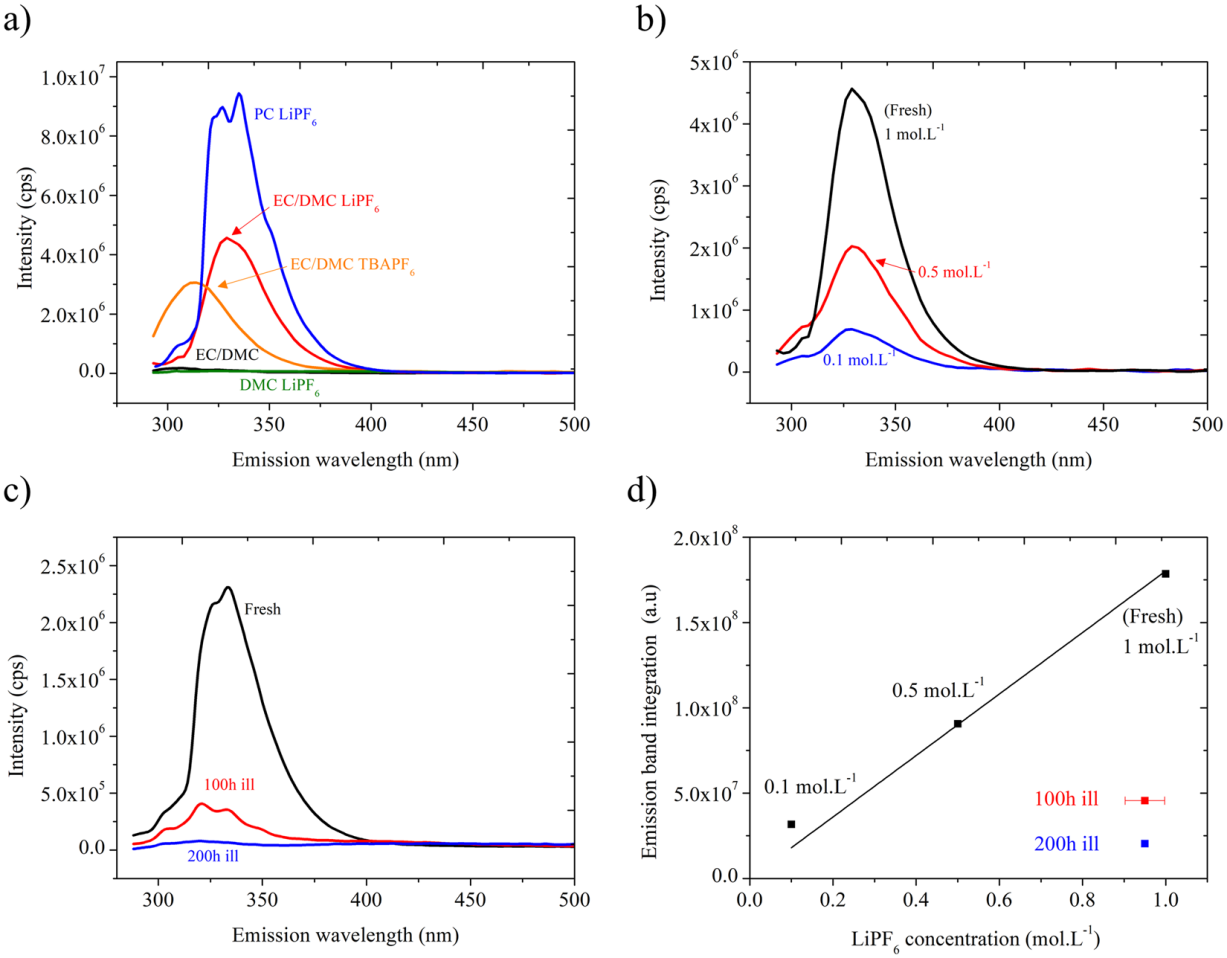
(**a**) Steady-state emission spectra for an excitation at 280 nm of EC/DMC in a 1/1 weight ratio (black curve), 1 mol.L^−1^ LiPF_6_ EC/DMC (red curve), 1 mol.L^−1^ TBAPF_6_ EC/DMC (orange curve), 1 mol.L^−1^ LiPF_6_ DMC (green curve) and 1 mol.L^−1^ LiPF_6_ PC (blue curve) (**b**) steady-state emission spectra of 1 mol.L^−1^ LiPF_6_ EC/DMC for different LiPF_6_ concentration in EC/DMC (1/1, w/w): 1 mol.L^−1^ (black curve), 0.5 mol.L^−1^ (red curve) and 0.1 mol.L^−1^ (blue curve) (**c**) Steady-state emission spectra of 1 mol.L^−1^ LiPF_6_ EC/DMC electrolyte for different illumination time: Fresh (black curve), after 100 hours (red curve) and after 200 hours illumination (blue curve) under A.M. 1.5G conditions (excitation wavelength: 280 nm). (**d**) 340 nm band integration values for the different LiPF_6_ concentration in EC/DMC. Red and blue spots stand for the integration value of the 100 hours and 200 hours illuminated sample, respectively.

This hypothesis is also consistent with our results on fluorescence spectroscopy, both in steady-state and time-resolved, for which such a spectroscopic tool is particularly sensitive to local environment. Steady-state fluorescence spectra, 2D excitation/emission maps and fluorescence quantum yield for the different electrolytes are presented in Figs 3c and S3, S4, respectively. For the fresh electrolyte, 2D exc./em. map exhibits only one contribution at 280 nm/330 nm (Figs 3c and S3). The absolute quantum yield value of fluorescence at an excitation of 280 nm, determined using an integration sphere with the most accurate direct/indirect excitation method to account for possible re-adsorption processes^24^, is ca. 7.7%. Thus, the main deactivation process of the excited-states proceeds through non-radiative paths (Fig. S4). To better decipher the specie(s) responsible for this luminescence, a series of solution have been investigated, namely pure DMC and pure EC/DMC mixture, DMC, EC/DMC and PC including 1 mol.L^−1^ LiPF_6_ and EC/DMC including 1 mol.L^−1^ of TBAPF_6_ (tetrabutyl ammonium hexafluorophosphate) (Fig. 3a). Unfortunately, 1 mol.L^−1^ LiPF_6_ in EC could not be measured accurately because of its large viscosity and the non-homogeneity of the mixture. For all the pure solvents and 1 mol.L^−1^ LiPF_6_ in DMC, the fluorescence yield is extremely weak. For this reason, the fluorescence stems from intermolecular deactivation of the excited-state between PF_6_^−^ and the cyclic EC solvent forming supramolecular structures. This is also supported by the linear relationship between PF_6_^−^ concentration and fluorescence yield (Fig. 3b,d). In addition, confirming the strong interplay between PF_6_^−^ and the cyclic solvent, a large increase of fluorescence intensity is experienced by replacing EC/DMC with PC. Conversely, a decrease of intensity associated with a hypsochromic shift to 312 nm is experienced by replacing Li^+^ by TBA^+^ in EC/DMC. The fact that solvation of PF_6_^−^ is mainly controlled by the cyclic EC or PC solvent is not surprising since their higher dielectric constant compared to the linear DMC (namely 3.10 F.m^−1^ for DMC, 64.92 F.m^−1^ for PC and 89.78 F.m^−1^ for EC)^25^. The blue-shift and weaker emission with TBA^+^ stems from a stronger screening of the charge delocalization in the excited states between PF_6_^−^ and EC.

For light-soaked electrolyte, a substantial change in electrolyte’s luminescence is experienced. It results in a decrease of luminescence after 100 hours, without any visible change in the related exc./em. band position. The quantum yield dropped to *ca*. 1.3%. The quenching process induced by light leads to a complete vanishing of luminescence after 200 hours of continuous exposure (Figs 3c and S3).

The solvation shell of lithium cation has been extensively studied to better understand the interplay between the electrolyte’s structure and the performances within lithium-ion batteries^26–30^. Nontheless, Ponnuchamy *et al*. on the basis of molecular dynamic calculations showed that the hexafluorophosphate anions contribute to the first solvation shell of lithium cation forming a Li^+^(EC)_2_(PF_6_^−^) complex in the case of EC/DMC binary systems^30^. The affinity of PF_6_^−^ with respect to cyclic carbonates stems from hydrogen bonding owing to the high electronegativity of the fluorinated anion^31^. Along the same line, more recently, Xu *et al*. highlighted the existence of a small population of PF_6_^−^ clusters with EC molecules (<1%) through hydrogen bonding based on Electrospray Ionization Mass Spectrometry (ESI-MS) and ^19^F NMR^32^. On the basis of these studies, we support that the fluorescence and its bleaching stems from such a supramolecular organization with deactivation of the excited-states through intramolecular charge transfer processes. The greater fluorescence in PC-based electrolyte can originate from modification of PF_6_^−^ affinity with PC compared to EC through the additional methyl group in the heterocycle. Light exposure thus induces a local reorganization between ions and solvent molecules leading to the breaking of the supramolecular structure by PF_6_^−^/EC interaction weakening. Note that an effect from a slight loss of PF_6_^−^ species as deduced by ERETIC-NMR can be excluded in this case as it deviates too excessively from linearity observed between PF_6_^−^ concentration and fluorescence yield (Fig. 3d).

**Figure 4:**
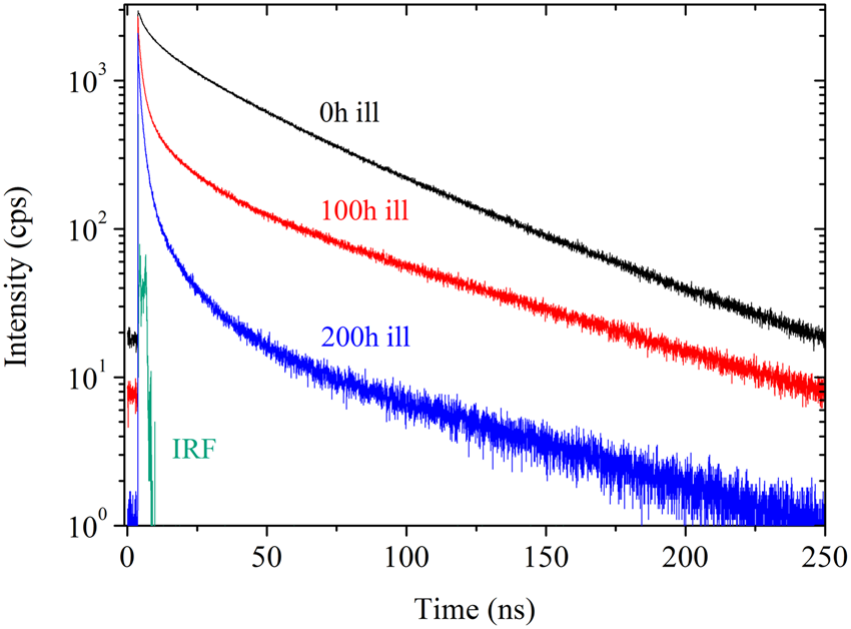
Fluorescence decay at excitation = 280 nm and emission = 330 nm for fresh 1 mol.L^−1^ LiPF_6_ EC/DMC electrolyte (black curve), after 100 hours (red curve) and 200 hours (blue curve) of light illumination under A.M. 1.5G conditions.

This reorganization at molecular level is also supported by Time-Correlated Single Photon Counting (TCSPC) experiments showing a modification of the average amplitude in the excited-state lifetime (Fig. 4). In this experiment, the excitation was set at 280 nm (polarized) and emission probed at 330 nm (at magic angle) with an accumulation time limited to 15 minutes to avoid long laser excitation. The excited-state lifetime was deduced after reconvolution from the instrumental response function (IRF ≈ 40 ps). Best reconvolution of the decays was obtained using three components. This suggests a complex radiative de-excitation process of PF_6_^−^/EC excited-states, involving a distribution of at least three different PF_6_^−^/EC intermolecular organizations that relax with a different time constant, possibly depending on the exciton radius in the cluster. The three lifetimes and amplitude average lifetime are tabulated in Table 2. As showed in Fig. 4, the new molecular organization obtained after illumination yields to a noticeable decrease in the average lifetime of the excited-state from 29.7 ± 0.7 ns for fresh electrolyte, to 9.9 ± 0.6 ns and 3.2 ± 2.5 ns after 100 and 200 hours of illumination. This originates from the greater dominance in the pre-exponential factor of the fastest component with illumination time (from 26.9 ± 10% for fresh electrolyte to 66.2 ± 6.5% and 80.4 ± 6.1% after 100 and 200 hours of light soaking). This suggests that light exposure prompts a loss of long-scale PF_6_^−^/EC organization. At the same time, this implies a greater proportion of excited species which are relaxing according to non-radiative paths.

**Table 2:** Evolution of fluorescence quantum yield and excited-state lifetimes of fresh 1 mol.L^−1^ LiPF_6_ EC/DMC electrolyte and after 100 and 200 hours of continuous light illumination under A.M. 1.5 G conditions.

	A_1_ (%)	τ_1_ (ns)	A_2_ (%)	τ_2_ (ns)	A_3_ (%)	τ_3_ (ns)	τ_ave_ (ns)	χ²	Quantum Yield (%)
Fresh	26.9 ± 10	3.1 ± 0.4	29 ± 3.1	18.4 ± 0.5	44.1 ± 0.9	53.2 ± 0.5	29.7 ± 0.7	1.015	7.7
100 h ill	66.2 ± 6.5	1.4 ± 0.1	21 ± 1.5	9.4 ± 0.5	12.7 ± 2	55.5 ± 1.2	9.9 ± 0.6	0.998	1.3
200 h ill	80.4 ± 6.1	1.2 ± 0.07	16.2 ± 7.4	6.3 ± 0.4	3.4 ± 6.1	36.1 ± 2.2	3.25 ± 2.5	0.929	≈0

The lifetime values were obtained by a multi-exponential decay after reconvolution of the IRF. Average lifetime was calculated using the amplitude of each components.

**Figure 5:**
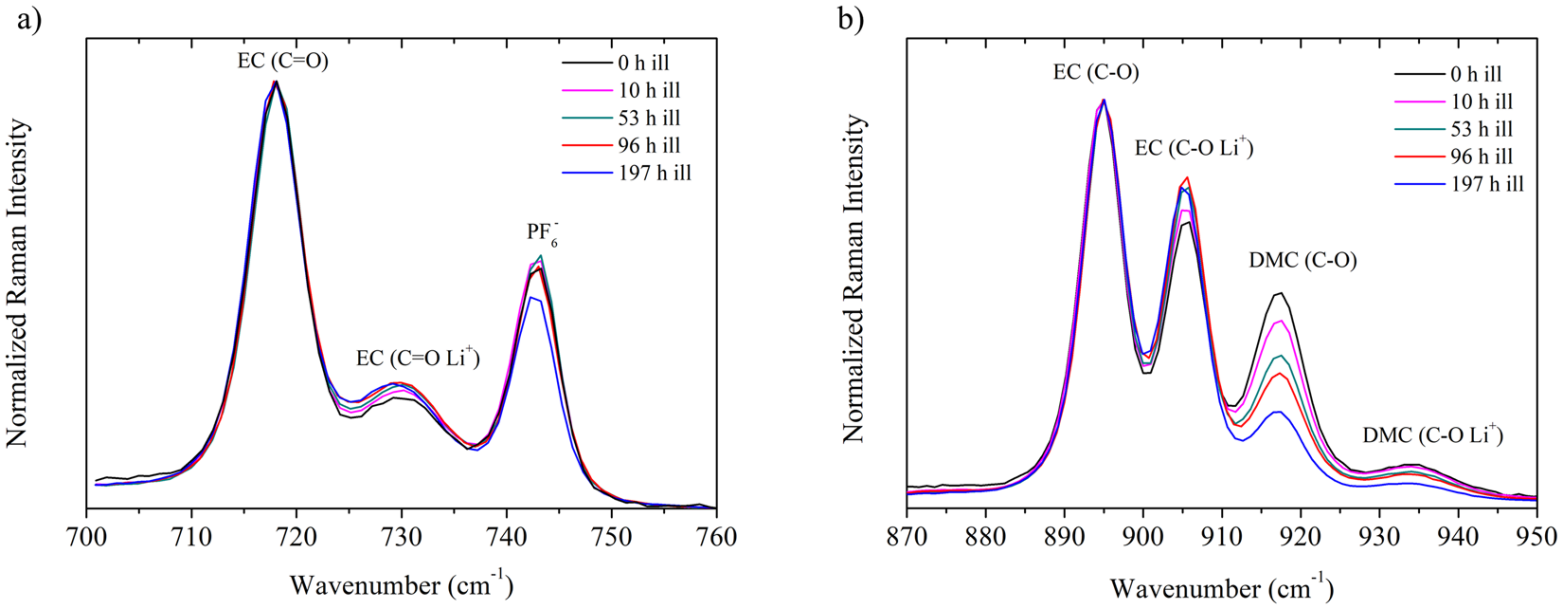
Evolution of Raman spectra of illuminated 1 mol.L^−1^ LiPF_6_ EC/DMC as a function of continuous illumination time (**a**) 700–760 cm^−1^ region and (**b**) 870–950 cm^−1^ region.

The modification in solvation strength and ion interactions in electrolyte have also been monitored by both Raman spectroscopy and Fourier-Transform InfraRed spectroscopy (FTIR) in ATR mode (Attenuated Total Reflectance). These technics are particularly powerful to probe with excellent accuracy molecular interactions in gas, solid and liquid phases. These vibrational spectroscopies have already been used for different kinds of battery electrolytes to get insights on the solvation of lithium in both linear and cyclic carbonated solvents with different counter-ions^26,29,32–38^. Figure 5a,b show the evolution of the Raman spectra upon continuous illumination up to 200 hours in the 700–760 cm^−1^ and 870–950 cm^−1^ regions which correspond to C=O and C-O vibrational modes, respectively^33–35,38^. For the 700–760 cm^−1^ region, the first band at 718 cm^−1^ is assigned to the free C=O bending mode related to ethylene carbonate^38^. The second and the third band at 730 cm^−1^ and 743 cm^−1^ corresponds to the C=O bending mode of EC in interaction with lithium cation^36,37^ and the vibrational mode of PF_6_^−^ anion, respectively^29,33,35,38^. Whereas these two latter show no clear energy shifts, it appears a modification in the band relative intensities compared to the free EC one, namely a gradual increase of the band at 730 cm^−1^ and a small decrease for the band at 743 cm^−1^ after 200 hours. The four bands in the second region (870–950 cm^−1^) are ascribed to the stretching mode of free C-O from EC (895 cm^−1^), C-O in interaction with lithium cation in EC (905 cm^−1^), free C-O from DMC (917 cm^−1^) and C-O in interaction with lithium cation in DMC (934 cm^−1^) (Fig. 5b)^33,35,38^. Interestingly, more important changes in relative intensities are experienced in this region without any substantial shifts in the band positions. As for the C=O bending mode, relative intensity of the lithium solvated bands with EC compared to the free C-O stretching band of EC experience a gradual increase. For both bulk and lithium solvated DMC C-O stretching band mode, we also noticed a decrease of the band intensity with the illumination time, accompanied by an increase of the relative band intensity of lithium solvated DMC compared to the free DMC band. As DMC evaporation can be ruled out due to the experimental conditions, one hypothesis would be that the amount of free DMC inside the electrolyte is decreasing upon illumination to the benefit of lithium solvated DMC.

**Figure 6:**
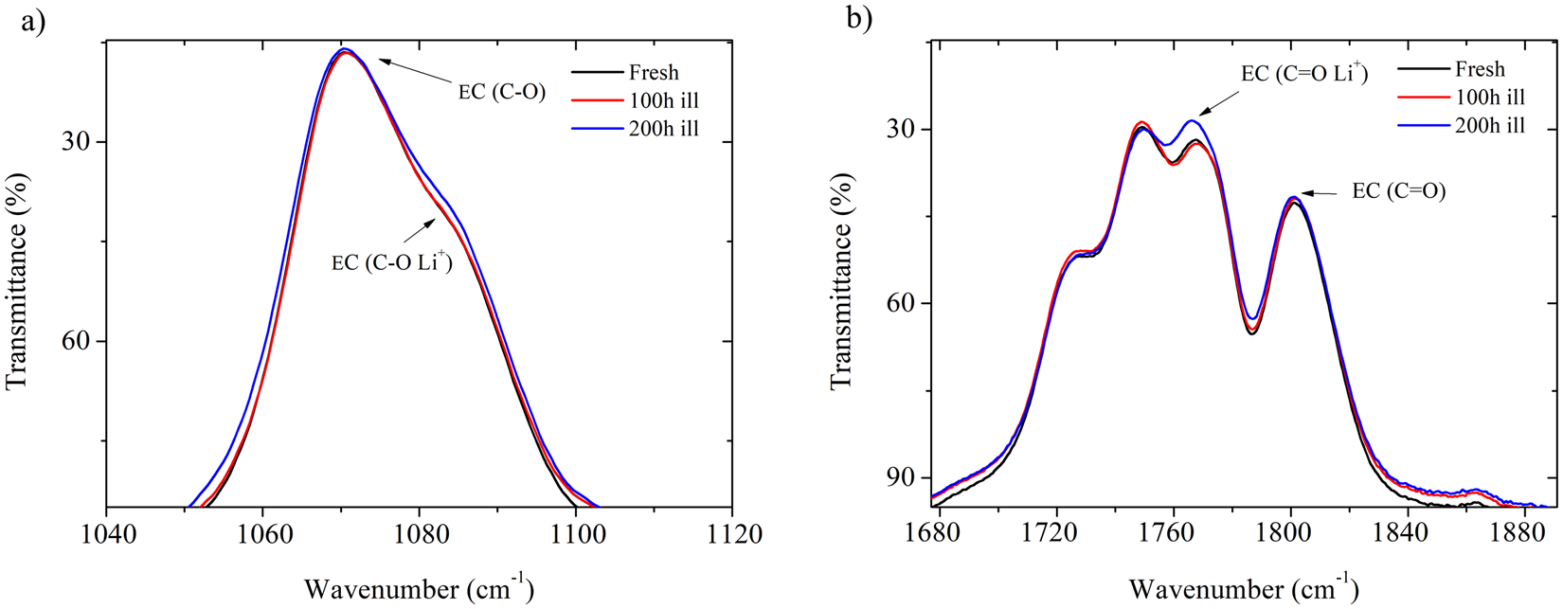
Evolution of FT-IR spectra recorded in ATR mode on fresh electrolyte based on 1 mol.L^−1^ LiPF_6_ in EC/DMC (black curve), after 100 hours (red curve) and 200 hours (blue curve) under A.M. 1.5G standard illumination (**a**) 1040–1120 cm^−1^ region and (**b**) 1680–1880 cm^−1^ region.

Similar observations on lithium solvation are made by FT-IR spectroscopy study in reflectance mode (ATR) (Fig. 6). It is showed that the bands related to the free molecules are unaffected by the light exposure by contrast to the solvent molecules in interaction with lithium cation which undergo noticeable intensity changes, in particular after 200 hours of light soaking. The broad band at 1070 cm^−1^ and its shoulder at 1085 cm^−1^ is ascribed to the C-O stretching mode of free EC and lithium-coordinated with EC through the C-O bond (Fig. 6a). C=O stretching modes are positioned at 1801 cm^−1^ for free EC and 1767 cm^−1^ for lithium-coordinated EC^39,40^ (Fig. 6b). As aforementioned, only weak changes are experienced after 100 hours by contrast to 200 hours light soaking. In good consistency with Raman spectroscopy, we are observing relative intensity modifications of the lithium-coordinated EC bands, namely a slight increase in intensity of the shoulder at 1085 cm^−1^ and at 1767 cm^−1^ (Fig. 6) as well as a small 1 cm^−1^ red-shift after 200 hours. Illumination thus modifies the way lithium is coordinated with the carbonated solvent molecules through the carbonyl group standing as the most electronegative part of the molecule^41^.

**Figure 7:**
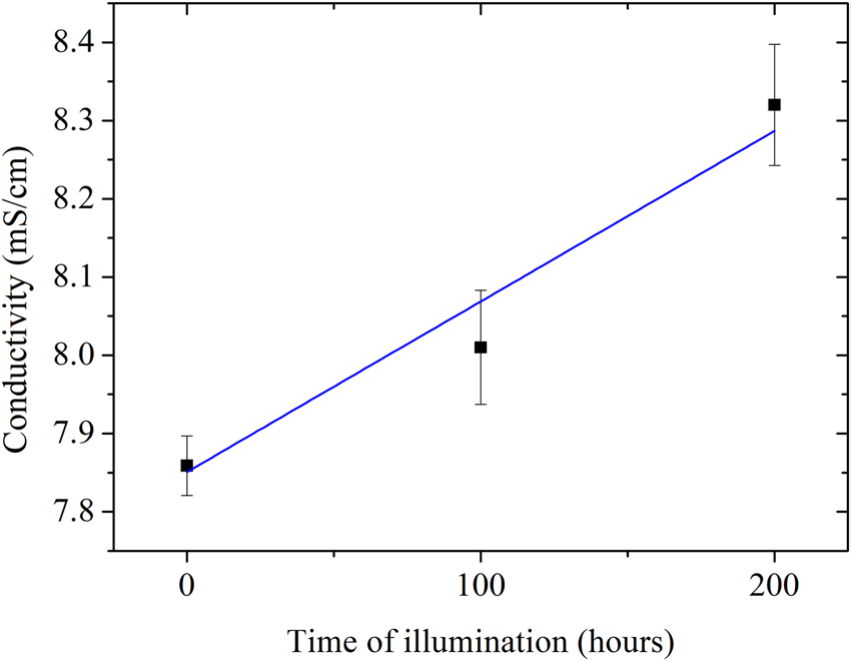
Room-temperature ionic conductivity measured by electrochemical impedance spectroscopy of fresh 1 mol.L^−1^ LiPF_6_ in EC/DMC electrolyte, after 100 hours and 200 hours of continuous light illumination under standard A.M. 1.5G conditions.

Modifications of relative band intensity have been reported on samples where the lithium concentration is modified^27,37^. Increasing lithium salt concentration leads to a decreasing in the intensity of the band related to free carbonyl while the one related to the carbonyl interacting with lithium cation is increasing. This intensity modification is explained by the formation of contact ion pair leading to a decrease in the lithium coordination number with the solvent. Our results based on both Raman and FT-IR spectroscopies show that prolonged light absorption by the electrolyte leads to an increase of the lithium coordination number with ethylene carbonate. This light-induced process should be likely very similar with other type of cyclic carbonates. This modification of lithium solvation upon illumination is the result from the supramolecular structure EC-PF_6_^−^ breaking subsequently to hydrogen bonding disruption. Although currently it is still unclear for us if prior illumination stage of the electrolyte has an impact or not on the electrochemical performances and electrolyte’s stability of lithium-ion batteries (we tested graphite/lithium in coin cell configuration), we found it has a positive impact on improving the electrolyte’s conductivity from 7.85 ± 0.038 mS.cm^−1^ for fresh electrolyte to 8.32 ± 0.077 mS.cm^−1^ after 200 hours of illumination (Fig. 7 and representative electrochemical impedance spectra in Fig. S5). Interestingly, ^31^P auto-diffusion NMR measurements were performed and highlighted that the diffusion coefficient of phosphor remains almost unchanged (D_fresh_ = 5.513 ± 0.07.10^−10^ m².s^−1^ vs D_100h_ = 5.559 ± 0.087.10^−10^ m².s^−1^). The amelioration of the ionic conductivity is hence governed by an enhancement related to the lithium cation in the electrolyte rather than the hexafluorophosphate anion.

## Conclusion

This study highlights the intrinsic stability of lithium-based electrolyte when exposed to continuous standard white light (A.M. 1.5G) based on NMR and GC/MS analysis. However, a set of different spectroscopic techniques, i.e. steady-state absorption and fluorescence, FT-IR and Raman spectroscopies, all together reveal for the first time that the electrolyte sustains a molecular re-organization through the solvation of Li^+^ and PF_6_^−^ around linear DMC and cyclic EC molecules. This new molecular organization leads to a slight yellowish coloration, improved electrolyte’s conductivity, complete quenching of luminescence and shortened excited-states lifetime from 29.7 ± 0.7 ns to 3.2 ± 2.5 ns.

## Methods

### Electrolyte

1 mol.L^−1^ EC/DMC electrolyte (battery grade) was purchased from Merck and used as received. The other electrolyte formulations were prepared using ethylene carbonate from Merck, dimethyl carbonate and propylene carbonate from Solvionic. LiPF_6_ and TBAPF_6_ salts were purchased from Sigma-Aldrich with battery grade.

### Illumination conditions

Electrolytes were placed in a quartz double-wall photo-electrochemical cell thermostated at 20 °C. Light exposure of the electrolyte was performed using a sun simulator LCS-100 94011A-ES from Oriel Instrument using a Xe lamp filtered A.M. 1.5G (1000 W/m^2^) with less than 5% spectrum mismatch to sunlight. These conditions correspond to photovoltaic standards. Close experimental conditions have been used for the Raman study using an airtight sealed quartz cell (1 cm length) to prevent DMC evaporation and LiPF_6_ degradation.

### NMR conditions

^1^H, ^19^F and ^31^P nuclear magnetic resonance (NMR) spectra were recorded on a Bruker DRX-400 spectrometer at 400, 376 and 162 MHz, respectively. Chemical shifts are reported in parts per million relatives to a residual solvent peak for CD_3_OD (^1^H: δ = 3.31 ppm). Samples were placed in a fluoropolymer liner in order to avoid glass degradation from LiPF_6_ degradation. The error bar for the concentration determination of PF_6_^−^ is determined based on the 5% sensitivity of NMR techniques. The one for the determined concentration (horizontal one) is calculated as 5% of the calibration curve slope.

### GC/MS conditions

The analyses were performed using a trace 1300 series GC ultra gas chromatograph (Thermo Scientific). The chromatographic separation was performed on a “BPX70” cyanopropyl polysilene–siloxane based capillary column (30 m × 0.25 mm i.d., 0.25 mm) from SGE. The GC was interfaced with an ISQ mass spectrometer (Thermo Scientific). Compound identification and corresponding structural formulae were assigned using the National Institutes of Standards (NIST) library.

### Steady-state fluorescence conditions

Fluorescence data were obtained using a FLS980 spectrometer from Edinburgh Instruments. Measurements were performed in a 4 faces polished quartz cuvette filled and sealed in Ar-filled glovebox (O_2_ and H_2_O level below 0.1 ppm). Excitation was ensured by a Xe lamp with two excitation monochromators scanning wavelengths from 250 to 474 nm with a slit aperture of 1 nm. Two emission monochromators fitted with a visible PMT detector probe the emission wavelengths from 262 to 500 nm with a slit aperture of 1 nm. Quantum yield measurements were performed on the same fluorimeter using a Benflec integration sphere. Excitation wavelength was set at 280 nm and the scans were recorded including both scattering and emission part in direct and indirect modes.

### TCSPC conditions

The fluorescence decays were measured by time-correlated single photon counting (TCSPC) technique. Excitation pulse was provided by a femtosecond Ti:sapphire laser (Coherent Chameleon Ultra II, 80 MHz, 200 fs, 3.8W) coupled to a pulse picker (4 MHz) and a harmonic generator (SHG/THG, APE) to get the 280 nm excitation. A set of dielectric mirror (250–320 nm reflexion) were used to direct (and remove the SHG and fundamental contribution after the harmonic generator) the 280 nm light into a FT200 Picoquant spectrometer (band pass 4 nm/mm). The emission was collected through a polarizer set at the magic angle and a Czerny-Turner type monochromator, computer-controlled for the selection of wavelength detection. The single-photon events were collected by a cooled microchannel plate photomultiplier tube R3809U (Hamamatsu) and recorded by a PicoHarp 300 TCSPC system (PicoQuant, 4 ps bintime). The instrumental response function was recorded using colloidal silica (Ludox), and its full width at half-maximum was in the range of 40 ps. The recorded decays were analyzed by FluoFit software package version 4.6.6 (PicoQuant). The reduced χ² was below 1.1. Weighted residuals and autocorrelation function were used to check the quality of the fits.

### ATR-FTIR conditions

ATR-FTIR measurements were performed using a Nicolet AVATAR 370 DTGS spectrometer (Thermo Scientific). A drop of electrolyte was deposited onto a diamond window and spectra were taken on 16 scans with a resolution of 1 cm^−1^. Background scans were performed prior to any measurements. The measurements were performed in a dry room with a dew point of around −55 °C to prevent any contribution of ambient moisture.

### Raman Spectroscopy conditions

Raman measurements have been performed using an air-tight seal flask in macro-Raman configuration using a Renishaw InVia spectrometer equipped with a Peltier cooled CCD detector. The 785 nm excitation line of a laser diode was used for the measurements.

### Conductivity measurements

Conductivity measurement were performed in Ar-filled glovebox with O_2_ and H_2_O level below 0.1 ppm using a platinum conductivity cell from radiometer analytical at room temperature. The error bar evaluation was determined by reproduction of the experiment over 5 measurements in which the greatest and lowest value has been removed.

## Electronic supplementary material

Supplementary information
