# Supplementary material for: Effect of standard light illumination on electrolyte’s stability of lithium-ion batteries based on ethylene and di-methyl carbonates
Source: Sci Rep. 2019 Jan 15;9:135. doi: 10.1038/s41598-018-36836-9 (PMC6333783; doi:10.1038/s41598-018-36836-9)
Supplement: Supplementary file 1 — Supplementary information [file 41598_2018_36836_MOESM1_ESM.pdf]

Effect of standard light illumination on electrolyte's stability of lithium-ion batteries based on ethylene and di-methyl carbonates

*Gaspard Bouteau[1-3], Albert Nguyen Van-Nhien[3], Michel Sliwa[4], Nicolas Sergent[5], Jean-Claude Lepretre[2,5], Grégory Gachot[1-2], Iryna Sagaidak[1,3], Frédéric Sauvage[1-2]**

[1]Laboratoire de Réactivité et Chimie des Solides, Université de Picardie Jules Verne (UPJV), CNRS UMR 7314, 33 rue Saint Leu, 80039 Amiens, France.

[2]Réseau sur le Stockage Electrochimique de l'Energie (RS2E), CNRS UMR 3459, France.

[3]Laboratoire des Glucides Université de Picardie Jules Verne (UPJV), CNRS UMR 7378, 33 rue Saint Leu 80039 Amiens, France.

[4] Univ. Lille, CNRS, UMR 8516 - LASIR - Laboratoire de Spectrochimie Infrarouge et Raman, F-59000 Lille, France.

[5] Laboratoire d'Electrochimie et Physicochimie des Matériaux et de Interfaces, Université de Grenoble Alpes, CNRS UMR 5279, 1130 rue de la piscine 38402 Saint Martin d'Hères, France

*E-mail : frederic.sauvage@u-picardie.fr

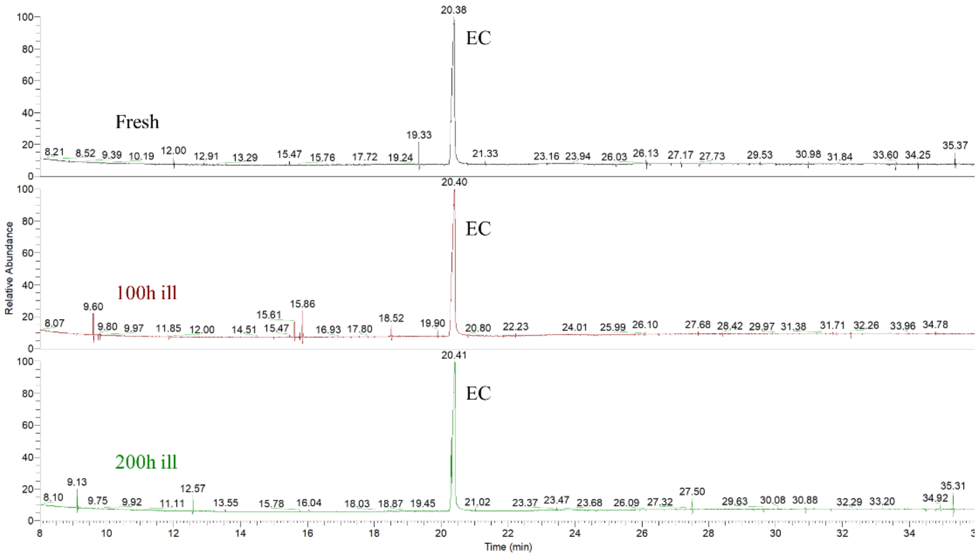

Figure S1 : GC/MS spectra for fresh 1 mol/L Li.PF_6 EC/DMC electrolyte and after 100 and 200 hours of light illumination under A.M. 1.5G conditions

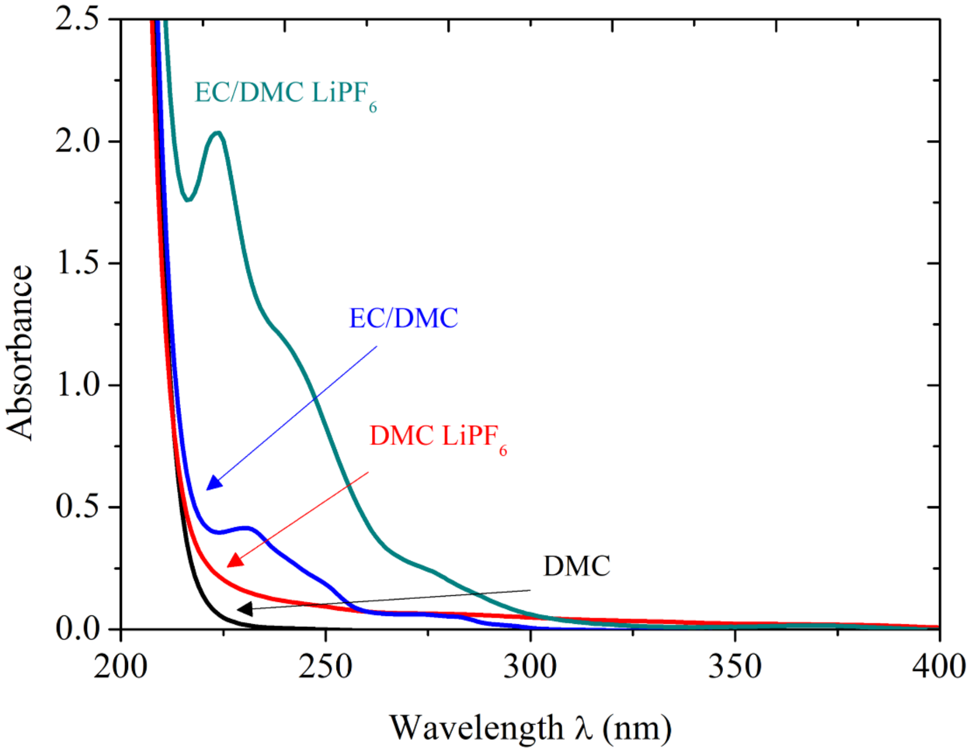

Figure S2 : UV-Vis absorption spectra of fresh 1 mol/L Li.PF_6 EC/DMC electrolyte and after 100 and 200 hours of light illumination under A.M. 1.5G conditions

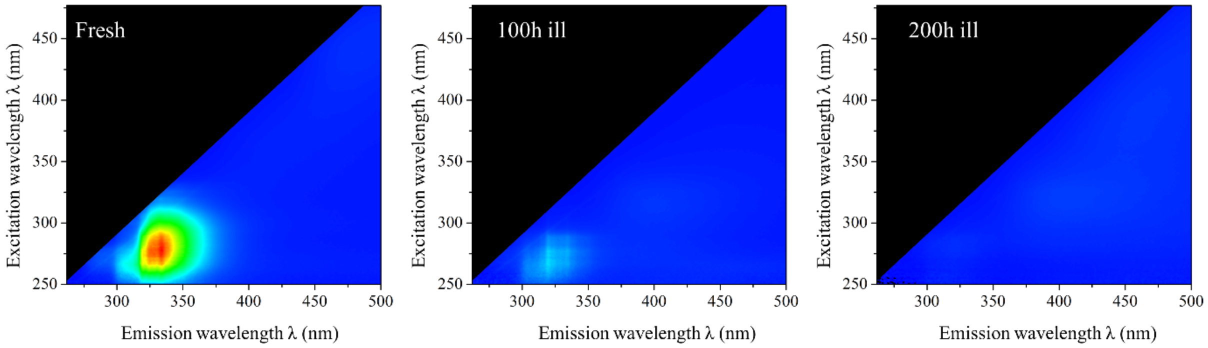

Figure S3 : Emission map for fresh 1 mol/L Li.PF_6 EC/DMC electrolyte and after 100 and 200 hours of light illumination under A.M. 1.5G conditions

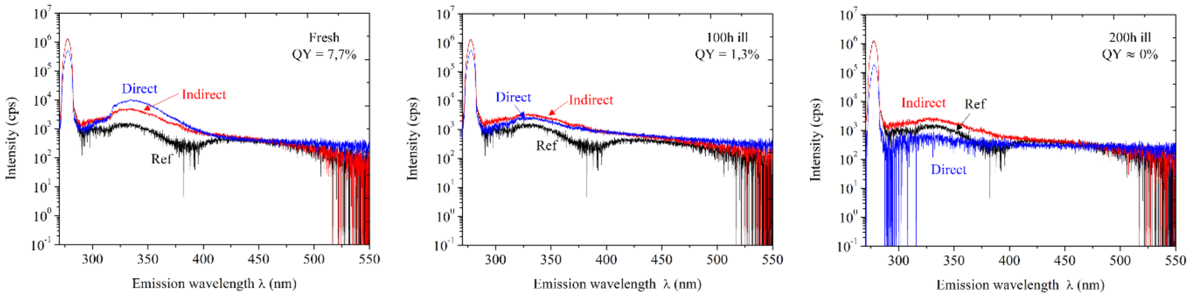

Figure S4 : Quantum yield spectra for fresh 1 mol/L Li.PF_6 EC/DMC electrolyte and after 100 and 200 hours of light illumination under A.M. 1.5G conditions

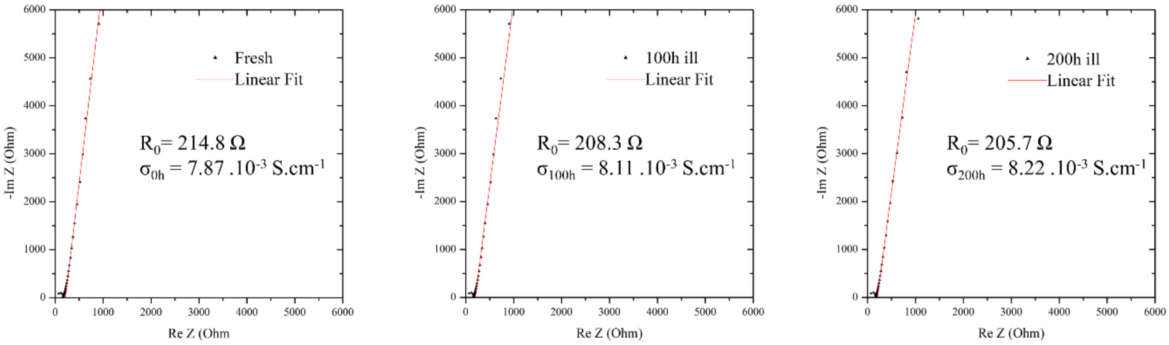

Figure S5 : Electrochemical impedance spectrum for fresh 1 mol/L Li.PF_6 EC/DMC electrolyte and after 100 and 200 hours of light illumination under A.M. 1.5G conditions
